# The interplay between kisspeptin and endocannabinoid systems modulates male hypothalamic and gonadic control of reproduction *in vivo*

**DOI:** 10.3389/fendo.2023.1269334

**Published:** 2023-10-12

**Authors:** Marianna Marino, Raffaella D’Auria, Elena Mele, Grazia Maria Giovanna Pastorino, Paola Di Pietro, Stefania D’Angelo, Natalia Della Rocca, Francesca Felicia Operto, Carmine Vecchione, Silvia Fasano, Riccardo Pierantoni, Andrea Viggiano, Rosaria Meccariello, Antonietta Santoro

**Affiliations:** ^1^ Dipartimento di Medicina, Chirurgia e Odontoiatria “Scuola Medica Salernitana” Università di Salerno, Baronissi, Italy; ^2^ Dipartimento di Scienze Motorie e del Benessere, Università di Napoli Parthenope, Napoli, Italy; ^3^ Unità Operativa Complessa (U.O.C.) Neuropsichiatria Infantile, Azienda Ospedaliero Universitaria San Giovanni di Dio Ruggi d’Aragona, “Scuola Medica Salernitana”, Salerno, Italy; ^4^ Dipartimento di Scienze della Salute, Università Magna Grecia di Catanzaro, Catanzaro, Italy; ^5^ Dipartimento di Medicina Sperimentale, Università della Campania L. Vanvitelli, Napoli, Italy

**Keywords:** Kisspeptin system, endocannabinoids, anandamide, cannabinoid receptors, hypothalamus, testis

## Abstract

**Introduction:**

Male reproduction is under the control of the hypothalamus–pituitary–gonadal (HPG) axis. The endocannabinoid system (ECS) and the kisspeptin system (KS) are two major signaling systems in the central and peripheral control of reproduction, but their possible interaction has been poorly investigated in mammals. This manuscript analyzes their possible reciprocal modulation in the control of the HPG axis.

**Materials and methods:**

Adolescent male rats were treated with kisspeptin-10 (Kp10) and endocannabinoid anandamide (AEA), the latter alone or in combination with the type 1 cannabinoid receptor (CB1) antagonist rimonabant (SR141716A). The hypothalamic KS system and GnRH expression, circulating sex steroids and kisspeptin (Kiss1) levels, and intratesticular KS and ECS were evaluated by immunohistochemical and molecular methods. Non-coding RNAs (i.e., *miR145-5p*, *miR-132-3p*, *let7a-5p*, *let7b-5p*) were also considered.

**Results:**

Circulating hormonal values were not significantly affected by Kp10 or AEA; in the hypothalamus, Kp10 significantly increased *GnRH* mRNA and aromatase Cyp19, Kiss1, and Kiss1 receptor (Kiss1R) proteins. By contrast, AEA treatment affected the hypothalamic KS at the protein levels, with opposite effects on the ligand and receptor, and SR141716A was capable of attenuating the AEA effects. Among the considered non-coding RNA, only the expression of miR145-5p was positively affected by AEA but not by Kp10 treatment. Localization of Kiss1+/Kiss1R+ neurons in the arcuate nucleus revealed an increase of Kiss1R-expressing neurons in Kp10- and AEA-treated animals associated with enlargement of the lateral ventricles in Kp10-treated animals. In the brain and testis, the selected non-coding RNA was differently modulated by Kp10 or AEA. Lastly, in the testis, AEA treatment affected the KS at the protein levels, whereas Kp10 affected the intragonadal levels of CB1 and FAAH, the main modulator of the AEA tone. Changes in pubertal transition-related miRNAs and the intratesticular distribution of Kiss1, Kiss1R, CB1, and CB2 following KP and AEA treatment corroborate the KS-ECS crosstalk also showing that the CB1 receptor is involved in this interplay.

**Conclusion:**

For the first time in mammals, we report the modulation of the KS in both the hypothalamus and testis by AEA and revealed the KP-dependent modulation of CB1 and FAAH in the testis. KP involvement in the progression of spermatogenesis is also suggested.

## Introduction

The hypothalamus–pituitary–gonadal (HPG) axis controls reproduction in both sexes and finds the main actors in the hypothalamic gonadotropin-releasing hormone (GnRH), pituitary gonadotropins, and gonadal sex steroids (1). Endocrine feedback mechanisms and also autocrine and paracrine communications along the HPG axis regulate reproductive functions in response to endogenous and exogenous environmental factors to guarantee the production of high-quality gametes (1). In males, the production of spermatozoa (SPZ) requires the coordination between mitosis, meiosis, and differentiation of germ cells inside the testis. All these processes are coordinated by an endocrine route involving the hypothalamic GnRH, the pituitary follicle-stimulating hormone (FSH), and the luteinizing hormone (LH). Gonadotropins target the somatic cells within the gonad (i.e., Sertoli cells and Leydig cells) which in turn produce sex steroids (i.e., mainly testosterone but also estradiol at a lesser amount) and sustain spermatogenesis progression to form high-quality SPZ.

The endocannabinoid system (ECS) and the kisspeptin system (KS) are two major signaling systems in the central and peripheral control of reproduction (2–8). The ECS comprises ligands [i.e., endocannabinoids like anandamide (AEA) and 2-arachidonoylglycerol (2-AG) and other endocannabinoid-like mediators such as N-acylethanolamines (NAEs)], canonical and non-canonical cannabinoid receptors [i.e., the G-coupled membrane receptors CB1 and CB2, the transient receptor potential cation channel subfamily V member 1 (TRPV1), and the nuclear peroxisome proliferator-activated receptors (PPARs) among others] (9), biosynthetic and hydrolyzing enzymes, and transporters (9). The KS currently includes kisspeptins, the cleavage products of the Kiss1 precursor, and the membrane kisspeptin receptor (Kiss1R) (5). In general, KS is considered the gatekeeper of the HPG axis from puberty onset onward (5) since central precocious puberty or hypogonadotropic hypogonadism occurs in humans and rodents as a consequence of gain of function and inactivating mutations of *KISS1/KISS1R*, respectively (10–12). Also, the ECS modulates the release of the hypothalamic GnRH, but its activity is opposite to KS. In fact, *in vivo* and *ex vivo*, the endocannabinoid AEA acts as retrograde signals to inhibit the release of GnRH (13–15). The characterization of the reproductive phenotype of CB1 knockout (CB1-/-) animals revealed the downregulation of the reproductive axis (16, 17). At the periphery, the ECS directly affects the postnatal development of Leydig cells (18, 19) steroidogenic ability (20), spermatogenesis progression (21, 22), and sperm maturation and quality (23–26). CBs are differently expressed in testicular germ cells and somatic cells in mammals (21, 22), and the modulation of endocannabinoid tone, primarily via the fatty acid amide hydrolase (FAAH), represents a key feature in endocannabinoid signaling. In general, CB1 has been linked to the control of the activity of Leydig cells (18–20), the recruitment of spermatogonial stem cells, chromatin remodeling, and the maturation of post-meiotic stages (22, 24, 26). Conversely, CB2 activity has been linked to the control of Sertoli cell physiology (27) and to spermatogenesis progression toward mitotic and meiotic stages (21). Endocannabinoid tone within the epididymis controls the acquisition of sperm motility in the cauda epididymis (23, 28), and data on CB1 pharmacological inhibition by its antagonist rimonabant (SR141716A) in human sperm and *CB1^−/−^* mice linked the inactivity of CB1 to reduced sperm survival and quality with defects in nuclear sperm size (25, 29) and disulfide bond formation (26). Involvement of the ECS in acrosome reaction and capacitation has also been reported in vertebrates including humans (2, 30).

Despite the well-known role of the KS in the central control of reproduction, at the periphery, the expression of the KS has been reported in cell lines, testis, SPZ, and epididymis collected from several species (31–44), but the real functions of the KS in the testis and SPZ are far to be fully elucidated (8, 45, 46). In general, Leydig cells and germinal epithelium are the main sources and targets of Kiss1 in several species (8), and Kiss1 secretion from testis has been suggested (47). However, due to different experimental designs, species, and methodologies, data reporting Kiss1/Kiss1R intratesticular localization, effects on steroidogenesis, spermatogenesis progression, and SPZ function are still debated (8, 45). Similarly, the involvement of the KS in Ca^2+^ mobilization and SPZ physiology has been investigated in humans and mice (36, 37) and is related to human sperm motility and hyperactivation (36) and fertilizing ability (37). Very recently, the involvement of Kiss1R in the cytoskeletal–nucleoskeletal pathway occurring in mouse Leydig cells to modulate gene expression was reported (40); in parallel, the possible role of the KS in epididymal SPZ maturation and storage was demonstrated in dogs (38) and rats (39), with significant Kiss1 levels measured within the epididymis (39). Based on the overall considerations, it appears that both ECS and KS centrally and locally regulate male reproduction; however, their possible interplay is still unraveled.

Hence, in this manuscript, we used an adolescent rat model treated *in vivo* with kisspeptin-10 (Kp10), or the main endocannabinoid AEA, alone or in combination with CB1 antagonist SR141716A, to investigate the possible reciprocal modulation of ECS and KS in rat testis. For the first time in mammals, we reported the modulation of the KS in both the hypothalamus and testis by AEA and revealed the KP-dependent modulation of CB1 and FAAH in the testis. The effects on the intratesticular distribution of Kiss1, Kiss1R, CB1, and CB2 following KP and AEA treatment are also reported.

## Materials and methods

### Chemicals and antisera

Metastin 45-54 of human origin (Kp10, h-YNWNSFGLRF-NH_2_) was provided by Santa Cruz Biotechnology, Inc., Milan, Italy (sc-221883). For *in-vivo* experiments, Kp10 was dissolved in saline at a concentration of 1 mg/ml, while N-arachidonylethanolamine (AEA, sc-396321A) and rimonabant [SR141716A, SR, N-(piperidino-1-yl)-5-(4-chlorophenyl)-1-(2,4dichlorophenyl)-4-methyl-pyrazole-3-carboxamide sc-205491A] (all by Santa Cruz Biotechnology, Herculer, California, USA) were dissolved in ethanol at a concentration of 20 mg/ml and 5 mg/ml, respectively. Stock solutions were further diluted in saline just before use as indicated below. A detailed list and the working conditions of the primary and secondary antisera used for Western blot, immunofluorescence (IFL), and immunohistochemistry (IHC) are given in **Table 1**.

**Table 1:** Primary and secondary antisera used for Western blot, IFL, and IHC.

Primary antisera in Western blot	Working dilution
Aromatase (Cyp19) anti-rabbit polyclonal IgG (E-AB-64300, Elabscience: Houston, TX, USA)	1:2000
CB1 (C-11) mouse monoclonal IgG (sc-518035, Santa Cruz Biotechnology)	1:350
CB2 (3C7) mouse monoclonal IgG (sc-293188, Santa Cruz Biotechnology)	1:3000
FAAH (27-Y) mouse monoclonal IgG_2_a (sc-100739, Santa Cruz Biotechnology)	1:500
3β-HSD (HSD3B1) rabbit polyclonal IgG (E-AB-15112, Elabscience)	1:700
Kiss1 (24-Q) mouse monoclonal IgG_2_a (sc-101246, Santa Cruz Biotechnology)	1:350
Kiss1R rabbit polyclonal IgG (E-AB-67516, Elabscience)	1:700
LIN-28 (6D1F9) mouse monoclonal IgG (sc-293120, Santa Cruz Biotechnology)	1:350
NAPE-PLD (E-8) mouse monoclonal IgG (sc-293120, Santa Cruz Biotechnology)	1:500
PCNA (PC10) mouse monoclonal IgG_2_a (sc-293120, Santa Cruz Biotechnology)	1:1000
Sirt-1 mouse monoclonal IgG1 (ab110304, Abcam: Cambridge, UK)	1:2000
α-Tubulin mouse monoclonal IgG (E-AB-20036, Elabscience)	1:5000
Secondary antisera in Western blot	
Goat-anti-rabbit IgG HPR (GtxRb003-DHRPX, ImmunoReagents: Raleigh, NC, USA)	1:4000
Goat-anti-mouse IgG HRP (GtxMu003-DHRPX, ImmunoReagents)	1:2000
m-IgGk-HPR conjugated (sc-516102, Santa Cruz Biotechnology)	1:2000
m-IgG-Fc-Bp-HRP conjugated (sc-525409, Santa Cruz Biotechnology)	1:500
Primary antisera in IFL and IHC	
CB1 rabbit polyclonal IgG (AB23703, Abcam)	1:100
CB2 rabbit polyclonal IgG (AB3561, Abcam)	1:200
Kiss1, clone 8H4.1 mouse monoclonal IgG1k (MABC60, Merck: Darmstadt, Germany)	1:50
Kiss1R rabbit polyclonal (BS-2501R, Bioss Antibodies, USA)	1:200 (IF) 1:50 (IHC)
NeuN rabbit monoclonal IgG (AB177487, Abcam)	1:500
Secondary antisera in IFL and IHC	
Horse-anti-rabbit IgG Dylight 488 (DI-1088, Vector Laboratories: Kirtlington, Oxfordshire, UK)	1:200
Horse-anti-mouse IgG Dylight 549 (DI-2549, Vector Laboratories)	1:200
Donkey-anti-rabbit IgG Biotin (DkxRt-003-FBIO, ImmunoReagents)	1:100

### Animals and tissue collection

Early peripubertal Wistar male rats (body weight 250–300 g) [38 post-natal days (pnd), *n* = 10/experimental group (Harlan Laboratories, Italy)] were housed two per cage with food and water available *ad libitum* and maintained on a 12-h light/dark cycle. Then, animals were divided into four groups identified using tail tags and treated as follows: group 1 control (saline 1% EtOH, C), group 2 KP (Kp10 0.1 mg/kg bw), group 3 AEA (2 mg/kg bw), and group 4 AEA (2 mg/kg bw) + SR (0.5 mg/kg bw). Treatments were performed two times a week. KP, AEA, and SR doses were chosen based on previously published works [KP (48, 49), AEA (24), and SR (23)]. Drugs were dissolved in a vehicle containing 99% saline and 1% ethanol, and SR was administered through intraperitoneal injection, whereas all the other animal groups received an equal volume of vehicle alone. Then after 30 min, rats were reinjected i.p. with the appropriate drug. After 3 weeks of treatment, rats were sacrificed by urethane (2 g/kg bw, Sigma-Aldrich, Milan, Italy) at 66 pnd (sexually mature). Blood was collected by cardiac puncture and processed for plasma collection. After being sacrificed, the testes and brain were removed; one testis/animal was frozen in liquid nitrogen and stored at −80°C until processing for RNA extraction and expression analysis by quantitative PCR (RT-qPCR) or Western blot; the other testis was fixed in Bouin’s fluids, embedded in paraffin following standard procedures, and then processed for morphological evaluation, IF, IHC, and Harris hematoxylin and eosin (H&E) staining. Brains were divided into two hemispheres that were randomly processed for molecular and morphological evaluations. In detail, one hemisphere of the brain was dissected to collect the mediobasal hypothalamus and immediately frozen in liquid nitrogen and stored at −80°C until processed for Western blot and qPCR; the contralateral hemisphere was fixed in 4% paraformaldehyde overnight and further processed for morphological evaluation, IF, and IHC. For IF and IHC, fixed brains were transferred to 70% ethanol, until they were included in paraffin. Serial coronal sections were sampled as already described (50) from bregma −2.04 mm to −5.04 mm according to the Paxinos and Watson rat brain atlas (51). The research protocol was approved by the Ethical Committee of the University of Salerno and the Italian Ministry of University and Research (authorization no. 66/2020-PR dated 29 January 2020).

### Gonadosomatic index and animal growth during the treatment

At sacrifice, the gonadosomatic index (GSI) was calculated using the formula:


[testes weight (g)/body weight (g) at sacrifice]×100

The growth of the animals was evaluated as:


100×[tibial lenght (mm)/body weight (g) at sacrifice]

### Plasma collection and measure of circulating Kiss1, testosterone, and estradiol

Blood was centrifuged at 800×*g* for 15 min at 4°C for plasma collection. Plasma was immediately frozen at −80°C until used for the measure of Kiss1 and sex steroid by enzyme-linked immunosorbent assay (ELISA).

The plasma levels of Kiss1 were measured by ELISA kit specifically designed for rat Kiss1 (CEC559Ra, Cloud-Clone Corp., Katy, TX 77494, USA) (50 µl/sample, *n* = 6 for the SR + AEA group; *n* = 7 for the control, KP, and AEA groups; intra-assay coefficient of variation<10%, interassay coefficient of variation<12%, detection range: 24.69–2,000 pg/ml, minimal detectable Kiss1 dose 9.22 pg/ml).

Plasma testosterone and estradiol levels, at basal conditions and following treatment, were measured using ELISA (Cat. No. DEV9911 and Cat. No. DEV9999, respectively; Demeditec Diagnostics GmbH, Germany). For the testosterone assay, 10 µl/sample was run in triplicate following the manufacturer’s instructions (*n* = 7; intra-assay coefficient of variation range, 6.50%–11.07%; interassay coefficient of variation range 9.3–11.3; detection range, 0.1–25 ng/ml; minimal detectable testosterone concentration, 0.066 ng/ml). For estradiol assay, 75 µl/sample was run in triplicate following the manufacturer’s instructions (*n* = 7; intra-assay coefficient of variation range, 3.0%–6.1%; interassay coefficient of variation range, 4.0%–7.1%; detection range, 5–1,280 pg/ml; minimal detectable estradiol concentration, 2.5 pg/ml).

All plates were read on a Bio-Rad plate reader (Bio-Rad, Milan, Italy), and data were calculated automatically using a 4-parameter logistics (4PL) curve fit (https://www.myassays.com/index.html).

### Total protein extraction and Western blot

Rat testes (*n* = 6) and hypothalamus (*n* = 4) from *in-vivo*-treated animals were processed for the extraction of total proteins by the RIPA Lysis Buffer System (Santa Cruz Biotechnology sc-24948) supplied with protease inhibitor cocktail (10 µl/ml of RIPA), 200 mM of phenylmethylsulfonyl fluoride (PMSF, 10 µl/ml of RIPA), and 100 mM of sodium orthovanadate (10 µl/ml RIPA) following the manufacturer’s instructions. Cleared protein extracts were collected by centrifugation at 11,000×*g* for 30 min at 4°C and quantified by Lowry assay (52).

Total proteins (40 µg) were resolved on precast 4%–20% Mini-Protean TxG gels (Bio-Rad Laboratories, CA, USA) and electrotransferred into PVDF filters by the TransBlot Turbo Transfer System (Bio-Rad Laboratories). Membranes were blocked in UltraCruz Blocking solution (sc-516214, Santa Cruz Biotechnology) for 1 h at room temperature (RT) and incubated overnight with primary antiserum (details in **Table 1**). After washing three times for 10 min in T-TBS (0.25% Tween-20 in Tris-buffered saline, TBS, pH 7.6) at RT, membranes were incubated 1 h at RT with the appropriate horseradish peroxidase-conjugated (HRP) secondary antiserum (details in **Table 1**) in UltraCruz Blocking solution and then washed again in T-TBS. Western blotting luminol reagent was used to detect the signals (Santa Cruz Biotechnology sc-2048). To quantify the protein signal, each membrane was stripped at RT for 30 min in stripping buffer (Santa Cruz Biotechnology sc-281698) and then reprobed with the mouse monoclonal anti-α-tubulin (details in **Table 1**). Autoradiographic films were scanned; signals were plotted and quantified with ImageJ. Western blot data are reported as specific protein optic density (OD)/housekeeping OD ± SD (*n* = 3 at least).

### RT-qPCR

Total RNA was extracted by TRIzol reagent (Thermo Fisher Scientific, Milan, Italy) following the manufacturer’s instructions and treated with DNAseI to eliminate any contamination of genomic DNA (Invitrogen Life Technologies: Carlsbad, California, USA). After quantification and quality evaluation on agarose gel, 2.5 µg of total RNA was reverse-transcribed in a final volume of 20 µl using SuperScript-III RnaseH^–^ Reverse Transcriptase (Thermo Fisher Scientific), 0.5 µg of oligo dT (18), 0.5 mM of dNTP mix, 5 mM of DTT, 1X first-strand buffer (Thermo Fisher Scientific), and 40 U of RNase Out (Thermo Fisher Scientific) following the manufacturer’s instructions. As a negative control, total RNA not treated with reverse transcriptase was used.

All real-time PCR assays were carried out in duplicate in the Mastercycler CFX-96 (Bio-Rad) using 1:2.5 diluted cDNA, 0.5 µM of each primer, and 10 µl of SYBR Green Master Mix (Bio-Rad) in a final volume of 20 µl. cDNAs were replaced by water in the negative controls. The primer sequences were as follows: *Aromatase*, *Cyp19*, forward, 5′-GCTTCTCATCGCAGAGTAT-3′, reverse, 5′-CAAGGGTAAATTCATTGGG-3′ (GenBank accession number: NM_017085); *GnRH*, forward, 5′-AGGAGCTCTGGAACGTCTGAT-3′, reverse, 5′-AGCGTCAATGTCACACTCGG-3′ (GenBank accession number: NM_012767); *3β-Hydroxysteroid dehydrogenase/Δisomerase (3βHsd)*, forward 5′-TCTACTGCAGCACAGTTGAC-3′, reverse 5′-ATACCCTTATTTTTGAGGGC-3′ (GenBank accession number: L17138); *β-Actin*, forward primer 5′-agatgacccagatcatgtttgaga-3′, reverse primer 5′-accagaggcatacagggacaa-3′ (GenBank accession number: NM_031144); or *hypoxanthine-guanine phosphoribosyltransferase (HPRT)*, forward primer 5′-TTGTTGGATATGCCCTTGACT-3′, reverse primer 5′-CCGCTGTCTTTTAGGCTTTG-3′ (GenBank accession number: NM_012583) used as endogenous control.

The 2^−ΔΔCt^ method was used for the relative quantification of gene expression (53). Assays also included a melting curve analysis for which all samples displayed single peaks for each primer pair. Data were reported as mean fold change ± SEM over the value one arbitrarily assigned to the control group.

### miRNA expression analysis

For the analysis of miRNA expression, miRNAs were extracted from the testis by the miRNeasy tissue/cells/advanced mini kit and cleared from DNA by the gDNA Eliminator Spin Columns (Qiagen, Milan, Italy). Following the manufacturer’s instructions, reverse transcription was carried out using the miRCURY LNA-RT Kit; qPCR was carried out in miRCURY LNA miRNA PCR Assay for the custom-validated primers for *hsa-miR145-5p*, *has-miR-132-3p*, *has-let7a-5p*, and *has-let7b-5p* and the housekeeping *U6* (reverse transcription kit, master mix, and validated primers all from Qiagen).

### Nissl staining of the hypothalamic nuclei and dimension of the lateral ventricle

Hypothalamus coronal sections were processed for Nissl staining as already described (54). Briefly, after deparaffinization and rinsing in distilled H_2_O, sections were incubated for 1 min in thionine. Slides were then dehydrated through serial ethanol solutions (70% to 100%), cleared in xylene, and mounted with Eukitt® Quick-hardening mounting medium. Images were acquired by using a bright-field microscope (Olympus SC180) at ×4 and ×10 magnification. Sections obtained from the left cerebral hemisphere were imaged for bright-field microscopy using the Olympus SC180 microscope. The areas of the lateral ventricle were manually traced and measured using ImageJ, and the measurements (*n* = 4 animals for each group) were recorded in square millimeters (54).

### IFL

For paraffin-embedded sections of the testis and hypothalamus, the IF protocol included deparaffination in Clear-Rite 3 (Thermo Scientific, Waltham, MA, USA) rehydration through descending degrees of alcohol up to water and non-enzymatic antigen retrieval in sodium citrate buffer 10 mM, pH 6.0, for 30 min at 95°C. Slides were washed three times with ice-cold PBS for 10 min and permeabilized with 0.1% Triton X-100 in PBS. For Kiss1 and Kiss1R analysis, a treatment with HCl 2 M for 30 min at 37°C was performed. Then, the slides were washed in PBS and blocked with a solution containing 10% of appropriate normal serum in PBS for 1 h at RT. The testis sections were incubated with anti-Kiss1, anti-Kiss1R, anti-CB1, and/or anti-CB2 or stained with 4′,6-diamidine-2′-phenylindole dihydrochloride solution (DAPI, 1.5 µg/ml in distilled H_2_O), while the hypothalamus sections were processed using anti-neuronal nuclei (NeuN) and anti-Kiss1. All sections were incubated with primary antisera overnight at 4°C in a humidified chamber. After three washing steps, the slides were incubated with the appropriate fluorescent dye-conjugated secondary antibodies for 1 h at RT. Details of the primary and secondary antisera are given in **Table 1**.

Negative controls were performed using all reagents except the primary antibody. Samples were then coverslipped with mounting gel (80% glycerol in PBS).

The samples were analyzed using a confocal laser scanning microscope (Leica SP5, Leica Microsystems, Wetzlar, Germany). Images were acquired in sequential scan mode by using the same acquisition parameters (laser intensities; gain photomultipliers; pinhole aperture; magnification ×20, ×40, or ×63). Figures were assembled using Adobe Photoshop 7 and Adobe Illustrator 10.

### IHC

Ten-micron-thick brain sections, mounted on poly-L-lysine-coated glass slides, were analyzed by IHC using the anti-Kiss1R antibody (details and working conditions in **Table 1**). The IHC protocol included deparaffination, non-enzymatic antigen retrieval, and permeabilization as reported in the previous section. Then, the slides were immersed in 3.6% hydrogen peroxidase solution for 10 min to inactivate endogenous peroxidases and blocked with a solution containing 10% of normal serum in PBS for 1 h at RT. After rinsing, the samples were incubated overnight at 4°C with anti-Kiss1R antibody. Then, the sections were washed twice in PBS and incubated with the secondary antibody for 1 h at RT, as in **Table 1**. Slides were then incubated with streptavidin-horseradish peroxidase (Vector Laboratories) for 1 h at RT. The immunoreaction signal was detected using a substrate chromogen solution containing 3,3-diaminobenzidine tetrahydrochloride (DAB substrate kit, Vector Laboratories). Finally, the sections were coverslipped with the Eukitt® Quick-hardening mounting medium (Sigma-Aldrich). For histological analysis, H&E staining was performed on the testis sections following standard procedures (54). Slides were analyzed using a brightfield microscope (Olympus SC180) at ×10 magnification. The number of the Kiss1R-positive cells within the arcuate nucleus (ARC) was counted in each section for *n* = 4 per experimental group using ImageJ software, and the mean number of cells/animal was used for further statistical analysis.

### Statistics

Student’s *t*-test and ANOVA followed by Duncan’s test were carried out to assess the significance of the differences in gene/protein expression, IFL, and IHC signal. Student’s *t*-test was used to assess the statistically significant differences between the treatment groups and the control group; ANOVA followed by Duncan’s test was used for multigroup comparison. Data were considered significant at *p<*0.05.

## Results

### Kp10 and AEA treatments do not affect the morphometric parameters of the treated animals

Weight gain is a recognized tool of puberty and SR has a possible anorectic effect (55). Hence, we measured the body weight and the length of the tibia to evaluate the correct growth of the treated animals vs. the control group during the 3-week-long treatments. At sacrifice, no differences in body weight, tibial length, testes weight, GSI, and growth were observed between the control and treated animals (**Table 2**).

**Table 2:** Morphometric parameters of the treated animals at sacrifice.

	Control (*n* = 10)	Kp10 (*n* = 10)	AEA (*n* = 10)	SR + AEA (*n* = 10)
**Body weight (g)**	327.5 ± 16.57	329.7 ± 23.04	319.4 ± 23.8	328.5 ± 31.5
**Tibial length (mm)**	44.8 ± 1.03	44.3 ± 1.25 *p* = 0.022 vs. SR + AEA	43.95 ± 1.92 *p* = 0.028 vs. SR + AEA	45.7 ± 1.25
**Testes weight (g)**	3.8 ± 0.19	3.55 ± 0.45	3.48 ± 0.36	3.48 ± 0.18
**GSI (%)**	1.035 ± 0.086	1.08 ± 0.089	1.09 ± 0.09	1.066 ± 0.09
Tibial length * 100/body weight	13.71 ± 0.74	13.51 ± 1.04	13.80 ± 0.77	14.01 ± 1.63

### Kp10 and AEA differentially modulated Kiss1R, Kiss1, and Cyp19 proteins*, GnRH* mRNA, and puberty-related miRs in the hypothalamus

**Figure 1:**
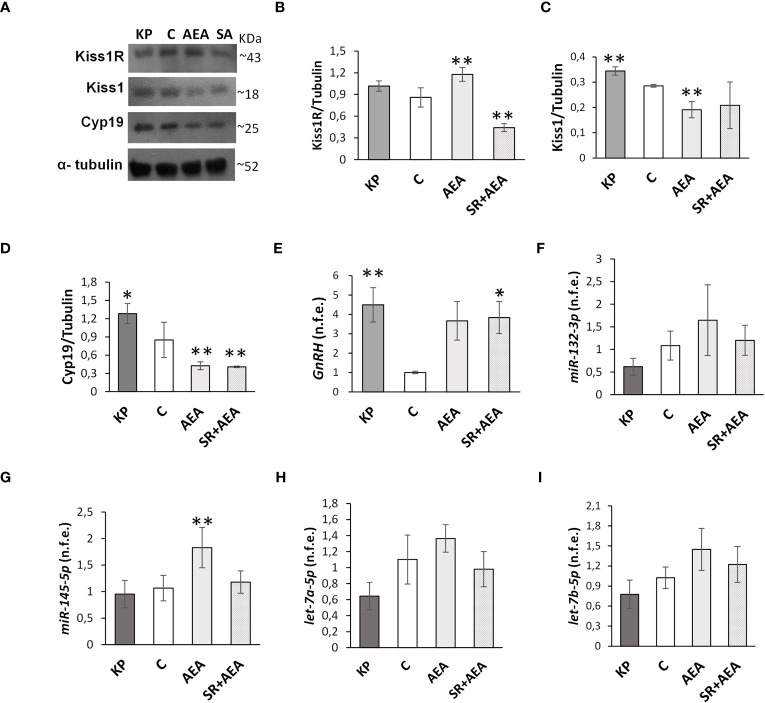
Modulation of Kiss1R, Kiss1, Cyp19, *GnRH*, and miR expression in mediobasal hypothalamus homogenates. Representative Western blots (*n* = 4) for Kiss1R, Kiss1, and Cyp19 **(A)** and normalization of Kiss1R **(B)**, Kiss1 **(C)**, and Cyp19 signals **(D)** carried out against α-tubulin. Quantitative expression (*n* = 6) of *GnRH* **(E)** and *miR-132-3p*, *Mir-145-5p*, *let-7a-5p*, and *let-7b-5p* **(F–I)** carried out by qPCR. Data are expressed as protein OD/tubulin OD ± standard deviation or normalized fold expression (n.f.e.) ± SEM against the housekeeping genes *HPRT*/*β-actin*/*U6* and the control group used as reference sample. C, control group (placebo injected animals); KP, animals injected with Kp10; AEA, animals injected with AEA; SR + AEA (SA), animals first received SR141716A and 30 min later received AEA treatment. **p< 0.01, *p< 0.05 vs. the control group.

Kisspeptin is the main upstream modulator of GnRH, from puberty onset onward (5); both kisspeptin and estradiol (5, 6, 31, 40), the sex steroid produced by the irreversible aromatization of testosterone by Cyp19 aromatase, were found as positive modulators of the KS. Conversely, the HPG axis is downregulated in CB1-/- mice (16, 17). To test the possible interplay between KS and ECS on the above issues, we evaluated the effects of Kp10 and AEA on Kiss1R, Kiss1, and Cyp19 proteins and *GnRH* mRNA in the mediobasal hypothalamus of the treated animals by Western blot and qPCR, respectively. The KP administration caused a slight increase of Kiss1R, Kiss1, and Cyp19 proteins and *GnRH* mRNA (**Figures 1A–E**), but the change was statistically significant only for Kiss1 and Cyp19 proteins and *GnRH* mRNA. AEA significantly increased Kiss1R protein (*p*< 0.01), decreased Kiss1 and Cyp19 proteins (*p*< 0.01), and increased *GnRH* mRNA, but the trend was not statistically significant (*p* = 0.065). Pretreatment with SR reverted the AEA-dependent effect on both Kiss1R and Kiss1 proteins but lowered Kiss1R protein below the control group, had no effect on Cyp19, and increased *GnRH* mRNA (*p*< 0.05).

Since treatments were carried out in pubertal animals and stopped at sex maturation, the effects of Kp10 and AEA treatments on the expression of miRs notably involved in the hypothalamic control of reproduction at puberty (56) were analyzed. The expression rate of *miR-132-3p*, *let-7a-5p*, *and let-7b-5p* did not change vs. the control group following KP or AEA treatment (**Figures 1F–I**); the expression of *miR-145-5p* was significantly higher in the AEA-treated group only and SR reverted this AEA-dependent effect (**Figure 1G**).

### Kp10 and AEA affect the number of Kiss1- and Kiss1R-expressing neurons in the hypothalamus, but only Kp10 increases the size of the lateral ventricle

**Figure 2:**
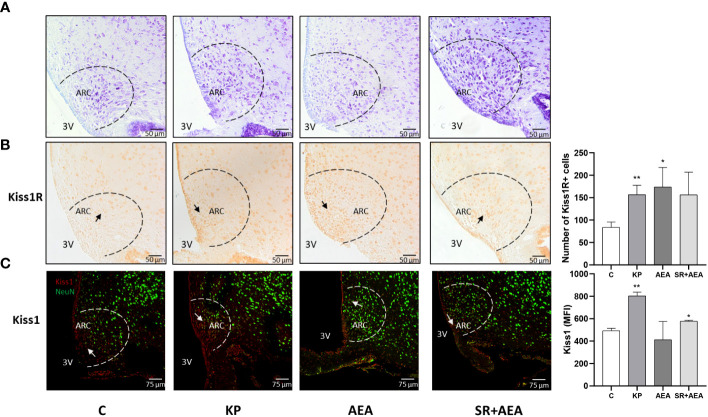
Expression and distribution of Kiss1R and Kiss1 proteins in the hypothalamic arcuate nucleus (ARC). Representative images (*n* = 4 for each animal group). Nissl staining showing ARC localization at ×20 magnification **(A)**; IHC of Kiss1R expression modulation in ARC, ×20 magnification **(B)**; IF analysis of the Kiss1 expression in ARC, ×40 magnification **(C)**. C, control; KP, animals injected with Kp10; AEA, animals injected with AEA; SR + AEA, animals first received SR141716A and 30 min later received AEA treatment. 3V, third ventricle. **p< 0.01; *p< 0.05.

Based on the Western blot results, we investigated whether the treatments specifically affected Kiss1 and Kiss1R proteins in the hypothalamic ARC, where GnRH is preferentially produced. Hence, we evaluated Kiss1 and Kiss1R in the ARC of treated and untreated rats (**Figures 2A–C**). Both KP and AEA treatments increased the number of Kiss1R-expressing neurons in the ARC compared with controls (*p*< 0.01 and 0.05, respectively) (**Figure 2B**), and the AEA-mediated effect was attenuated by SR administration. KP administration and the combined AEA/SR treatment increased the number of Kiss1-expressing neurons in the ARC (*p*< 0.01 and *p*< 0.05, respectively) (**Figure 2C**).

**Figure 3:**
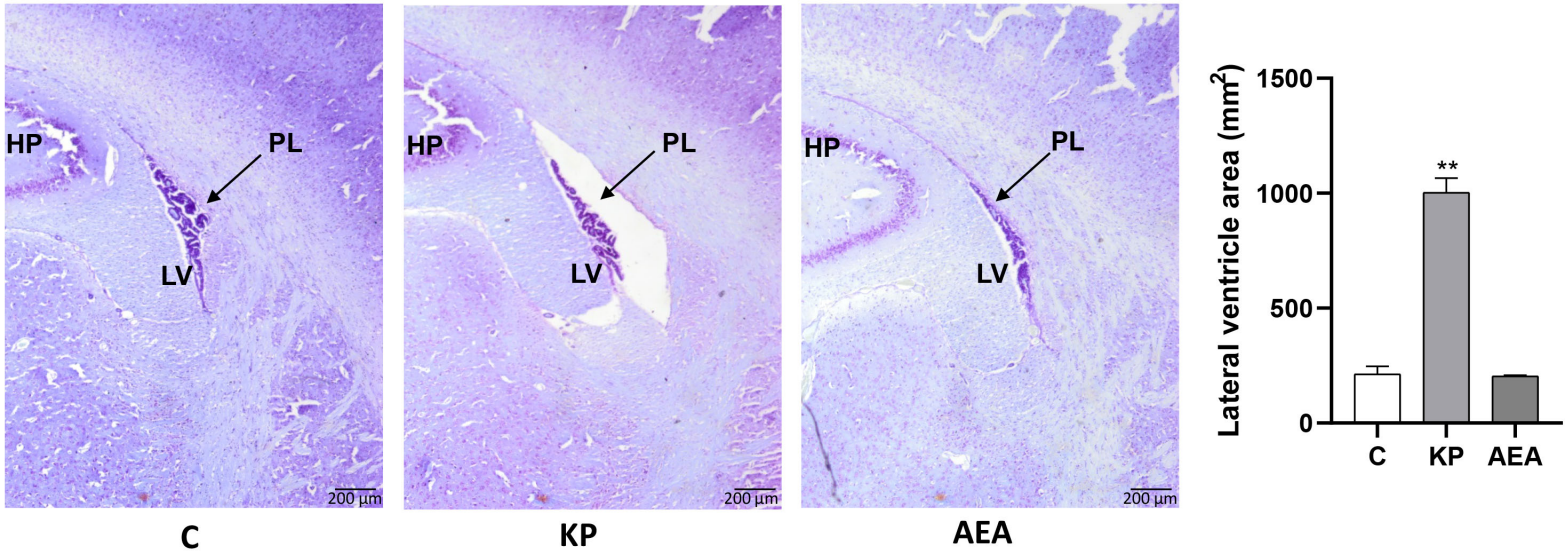
Morphological evaluation of the lateral ventricle. Representative images (*n* = 4 for each animal group) of Nissl staining showing the lateral ventricle of the control and treated animals. C, control; KP, animals injected with Kp10; AEA, animals injected with AEA; SR + AEA, animals first received SR141716A and 30 min later received AEA treatment. HP, hippocampus; LV, lateral ventricle; PL, choroid plexus. Magnification ×4. **p< 0.01.

Interestingly, Kiss1 was present not only within the ARC but also along the ependyma of the lateral ventricle as shown in the representative image of the control in **Figure 2C**. The KP treatment exacerbated this condition suggesting a role for this protein also in cerebrospinal fluid homeostasis (**Figure 2C**). To further investigate this issue, we analyzed the morphology and size of the lateral ventricle in KP-treated animals. A significant expansion of the ventricle area was observed compared with controls (*p*< 0.01) (**Figure 3**).

### The administration of Kp10 and AEA does not affect circulating hormonal levels

Changes in the circulating gonadotropins and sex steroids in turn are the main consequences of the overstimulation or inhibition of the HPG axis (1), and the possible effects of KP and AEA administration on the circulating levels of Kiss1 have never been investigated before in rodents. Thus, we measured Kiss1 and sex steroids in the blood to ascertain to what extent treatments affected the physiology of the HPG axis.

The circulating levels of Kiss1, testosterone, and estradiol were measured by ELISA following the *in-vivo* treatment with KP, AEA, and SR + AEA (**Table 3**) revealing the lack of any statistically significant change in the measured hormonal levels.

**Table 3:** Circulating hormonal levels.

	Control (*n* = 7)	Kp10 (*n* = 7)	AEA (*n* = 7)	SR + AEA (*n* = 7)
**Kiss1 (pg/ml)**	171.24 ± 47.5	235.05 ± 124.9	165.16 ± 54.2	212.86 ± 79.9
**Testosterone (ng/ml)**	2.46 ± 0.8	2.92 ± 2.55	2.24 ± 1.24	3.22 ± 1.47
**Estradiol (pg/ml)**	20.0 ± 6.37	24.5 ± 9.2	21.9 ± 4.7	27.3 ± 4.1

### Kp10 decreases *Cyp19* and *GnRH* mRNA in the testis

**Figure 4:**
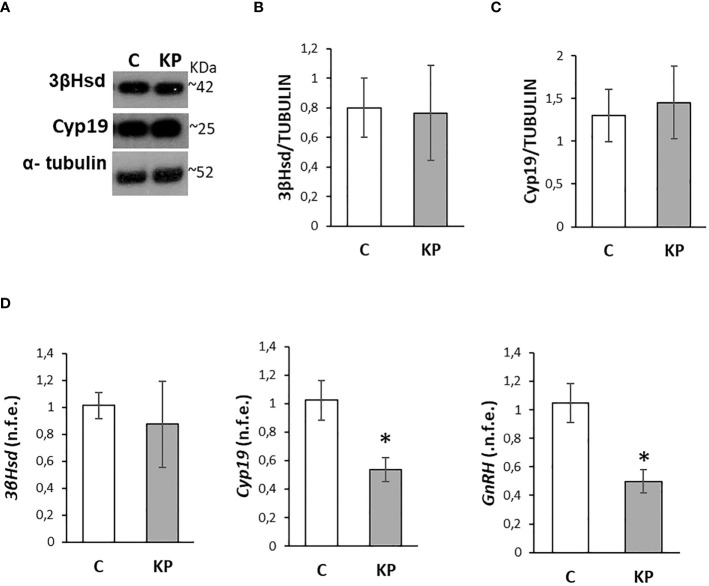
Modulation of the intratesticular markers of steroidogenesis and *GnRH*. Representative Western blots for 3βHsd and Cyp19 **(A)** and normalization of 3βHsd **(B)** and Cyp19 **(C)** signals carried out against α-tubulin. Quantitative expression of *3βHsd*, *Cyp19*, and *GnRH* mRNA carried out by qPCR **(D)**. Data are representative of *n* = 6 different animals and are expressed as protein OD/tubulin OD ± standard deviation or normalized fold expression (n.f.e.) ± SEM against the housekeeping genes *HPRT*/*β-actin* and the control group used as a reference sample. C, control group (placebo-injected animals); KP, animals injected with Kp10; *p< 0.05.

To further confirm the functionality of the HPG axis and the lack of significant effects on steroid biosynthesis, we investigated by Western blot the effects of the treatments on 3βHsd and Cyp19, the two main enzymes involved in testosterone biosynthesis and conversion into estradiol (57). In addition, the expression rate of intratesticular *GnRH* mRNA, involved in the control of the physiology of both Leydig cells and spermatogenesis (57), was also considered. Results revealed the absence of any statistically significant difference between the control group and the KP-treated group (**Figures 4A–C**). The trend of the corresponding mRNA was toward a decrease in the KP group for both *3βHsd* and *Cyp19* (**Figure 4D**) but was statistically significant only for *Cyp19* mRNA (*p*< 0.05 vs. the control group). The intratesticular expression of *GnRH* mRNA was significantly lower in the KP-treated group (*p*< 0.05).

### Effects on spermatogenesis progression: Kp10 increases Lin28A and Sirt1 proteins but has opposite effects than AEA on puberty-related miRs

**Figure 5:**
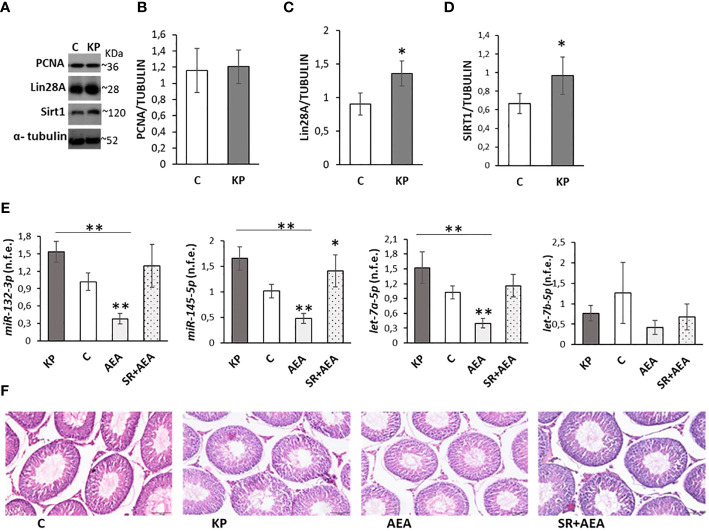
Testicular markers of proliferation, damage, and puberty onset. Representative Western blots (*n* = 6) for PCNA, Lin28a, and Sirt1 **(A)** and normalization of PCNA **(B)** Lin28A **(C)**, and Sirt1 signals **(D)** carried out against α-tubulin. qPCR (*n* = 4) for *hsa-miR145-5p*, *has-miR-132-3p*, *has-let7a-5p*, and *has-let7b-5p* **(E)**. Representative images of testicular morphology by H&E staining, ×10 magnification (*n* = 4) **(F)**. Data are expressed as protein OD/tubulin OD ± standard deviation in Western blot and as normalized fold expression (n.f.e.) ± standard deviation against the housekeeping gene *U6 in* qPCR (reference sample arbitrarily assigned 1 to the control group). C, control group (placebo-injected animals); KP, animals injected with Kp10; AEA, animals injected with AEA; SR + AEA, animals first received SR141716A and 30 min later received AEA treatment. *p< 0.05; **p< 0.01.

Due to the possible acceleration of spermatogenesis and tissue damage related to prolonged KP treatments (58, 59), molecular markers of spermatogenesis and testis morphology were investigated. The protein levels of molecular markers of the S phase of the cell cycle (i.e., PCNA) (60), spermatogenesis progression at puberty (i.e., Lin28A) (61), and cell survival (i.e., Sirt1) (62) were analyzed by Western blot (**Figure 5A**). The protein level of PCNA did not differ between the control and the KP-treated group (**Figure 5B** **)**, while both Lin28A and Sirt1 protein levels increased following KP treatment (*p*< 0.05) (**Figures 5C, D**).

The expression rate of miRNAs notably involved in the control of spermatogenesis at puberty (i.e., *miR145-5p*, *miR-1323p*, *let7a-5p*, *let7b-5p*) (61) was also analyzed by qPCR following KP and AEA treatments (**Figure 5E**). The expression rate of *miR145-5p*, *miR-1323p*, and *let7a-5p* was significantly lower in AEA-treated animals (*p*< 0.01 vs. the control group and vs. the KP group). The expression rate of *miR-132-3p* and *let7a-5p* was significantly higher in the KP-treated group (*p*< 0.05 vs. control), and the expression rate of *let7b-5p* did not change in all treatments. Pretreatment with SR reverted the AEA-dependent effects on *miR145-5p*, *miR-132-3p*, and *let7a-5p.*

Testis morphology in the control and treated animals was comparable (**Figure 5F**).

### Kp10 increases and AEA reduces the expression of Kiss1R in the testis

**Figure 6:**
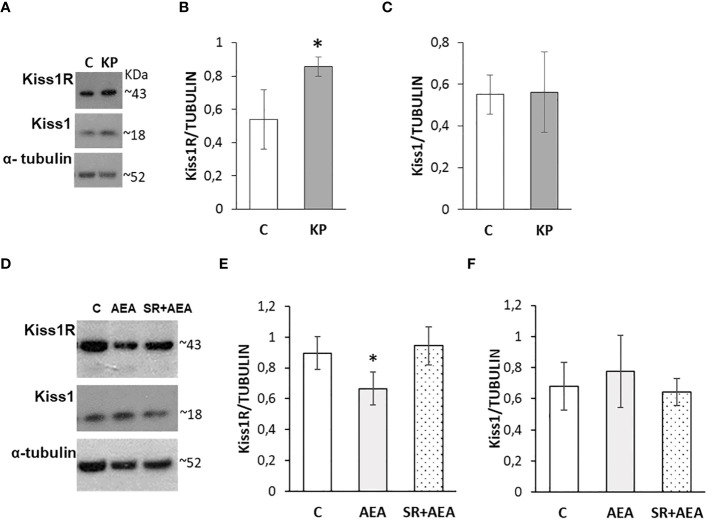
Modulation of the KS by KP **(A–C)** and AEA **(D–F)** in rat testis. Representative Western blots for Kiss1R and Kiss1 **(A, D)** and normalization of Kiss1R **(B, E)** and Kiss1 signals **(C, F)** carried out against α-tubulin. *N* = 6 different animals in KP treatments and *n* = 4 in AEA treatments; data are expressed as protein OD/tubulin OD ± standard deviation. C, control group (placebo-injected animals); KP, animals injected with Kp10; AEA, animals injected with AEA; SR + AEA, animals first received SR141716A and 30 min later received AEA treatment. *p< 0.05.

The possible self-modulatory loop of KP on the intratesticular KS was analyzed by Western blot (**Figure 6A**). The KP treatment significantly increased the protein level of the corresponding receptor (**Figure 6B**) (*p*< 0.05), without any significant effect on the ligand (**Figure 6C**).

Following the *in-vivo* treatment with AEA ± SR141716A, Kiss1R and Kiss1 proteins were analyzed by Western blot (**Figure 6D**). The AEA treatment significantly reduced the protein level of Kiss1R (**Figure 6E**) but did not have any significant effect on Kiss1 (**Figure 6F**) although an increasing trend could be observed. Pretreatment with SR141716A fully reversed the AEA-dependent effects on Kiss1R.

### Kp10 and AEA treatments differentially change the intratesticular localization of Kiss1 and Kiss1R

**Figure 7:**
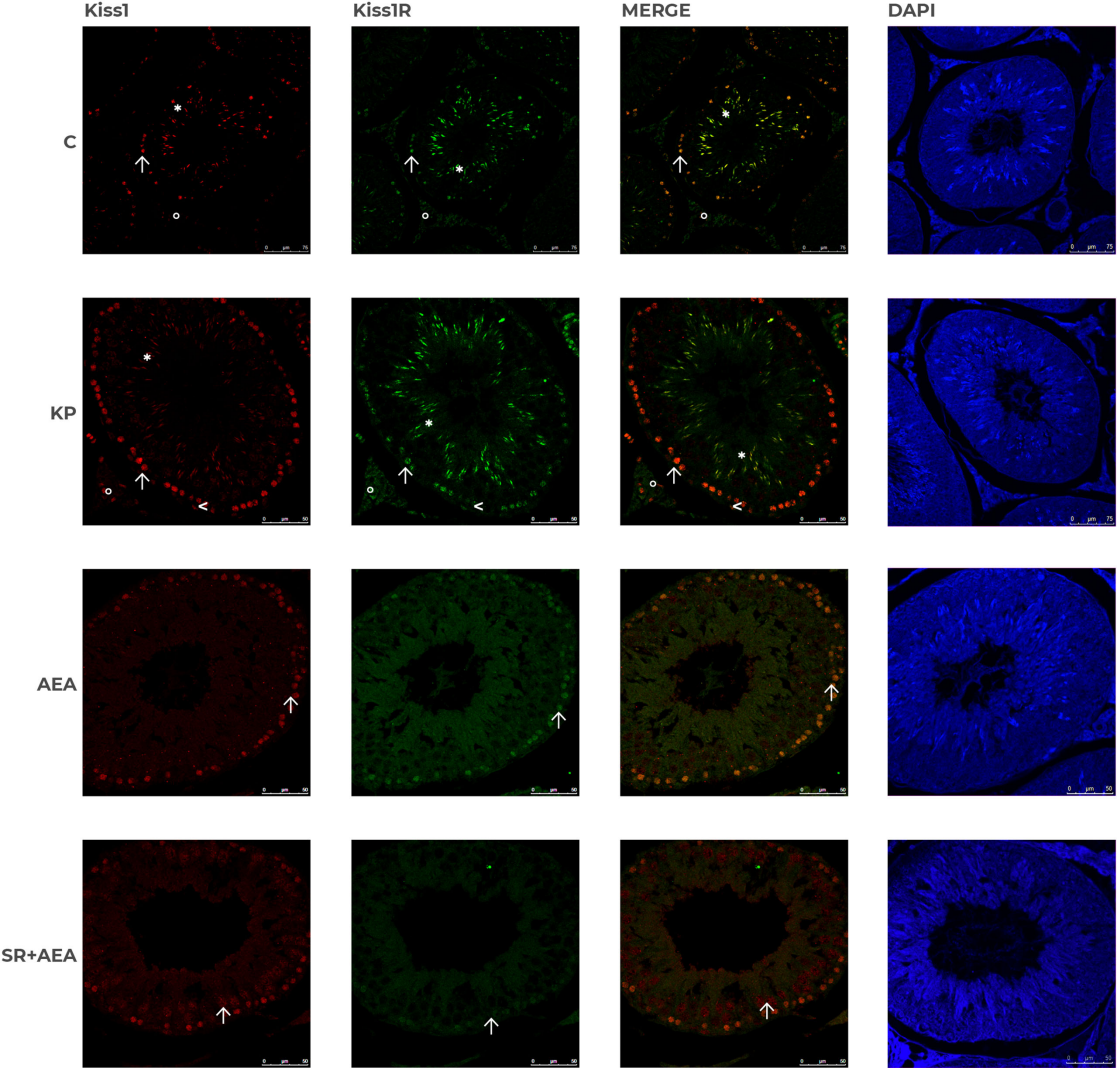
Distribution of Kiss1 and Kiss1R in rat testis treated with KP and AEA. Double immunofluorescence of Kiss1 (red) and Kiss1R (green) expression in control (C), kisspeptin (KP), anandamide (AEA), and SR141716A (SR + AEA)-treated animals (*n* = 4 for each group). The last column shows the representative images of DAPI staining on the same animals. Magnification = C ×40; KP, AEA, and SR+ AEA, ×63. * Elongated spermatids, ↑ spermatocytes, ^ spermatogonia, ° interstitium.

In control animals, the intratesticular localization of Kiss1 and Kiss1R was quite overlapping within the germinal epithelium (**Figure 7**). It marked mostly elongating spermatids close to the lumen and, to a lesser extent, premeiotic stages (i.e., preleptotene spermatocytes) close to the basal compartment within the germinal epithelium. In the interstitial compartment, a few Kiss1 and numerous Kiss1R-positive elements were detectable. Following the KP treatment, more intense staining was observed for both Kiss1 and Kiss1R in the interstitial compartment and the germinal epithelium. In detail, within the germinal epithelium, the immunoreactivity of Kiss1 and Kiss1R still characterized elongating spermatids but was also detectable in spermatocytes and a few spermatogonia. Following the AEA treatment, Kiss1 and Kiss1R localization in the spermatids was limited to a few scattered spots but was still evident in spermatocytes (**Figure 7**). A stronger staining for Kiss1 and Kiss1R was detectable in the SR + AEA-treated animals in both preleptotene and pachytene spermatocytes.

### Kp10 increases CB1 and FAAH proteins

**Figure 8:**
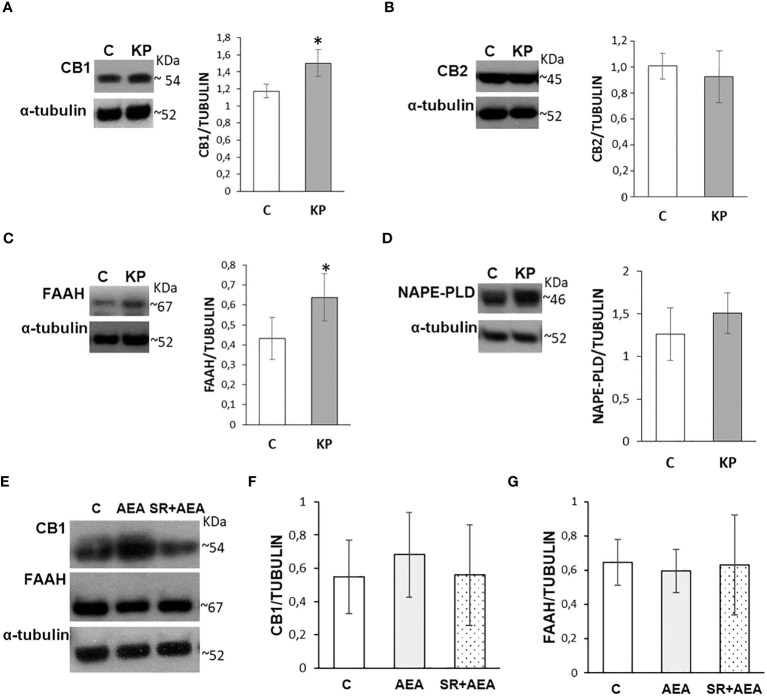
Modulation of the ECS by KP **(A–D)** and AEA **(E–G)** in rat testis. Representative Western blots and normalization of the signals carried out against α-tubulin. *n* = 6 different animals in KP treatments and *n* = 4 in AEA treatments. Data are expressed as protein OD/tubulin OD ± standard deviation. C, control group (placebo-injected animals); KP, animals injected with Kp10; AEA, animals injected with AEA; SR + AEA, animals first received SR141716A and 30 min later received AEA treatment. *p< 0.05.

The possible effects of *in-vivo* administration of KP on the testicular ECS were evaluated by Western blot focusing on the main cannabinoid receptors, CB1 and CB2, on FAAH and N-acylphosphatidylethanolamine-phospholipase D (NAPE-PLD), the main enzymes responsible for AEA degradation and biosynthesis, respectively. KP treatment significantly increased the protein level of CB1 (**Figure 8A**) (*p*< 0.05) but did not have any effect on CB2 (**Figure 8B**). Kp10 treatment significantly increased the protein level of FAAH (**Figure 8C**) (*p*< 0.05), without any statistically significant effect on NAPE-PLD (**Figure 8D**).

Since CB1 and FAAH were responsive to the KP treatment, a possible self-modulatory loop of AEA on its canonical receptor and its hydrolyzing enzyme was analyzed by Western blot following the AEA treatment (**Figure 8E**); a lack of any statistically significant change in CB1 and FAAH proteins was observed (**Figures 8F, G**).

### Kp10 and AEA treatments differentially change the intratesticular localization of CB1 and CB2

**Figure 9:**
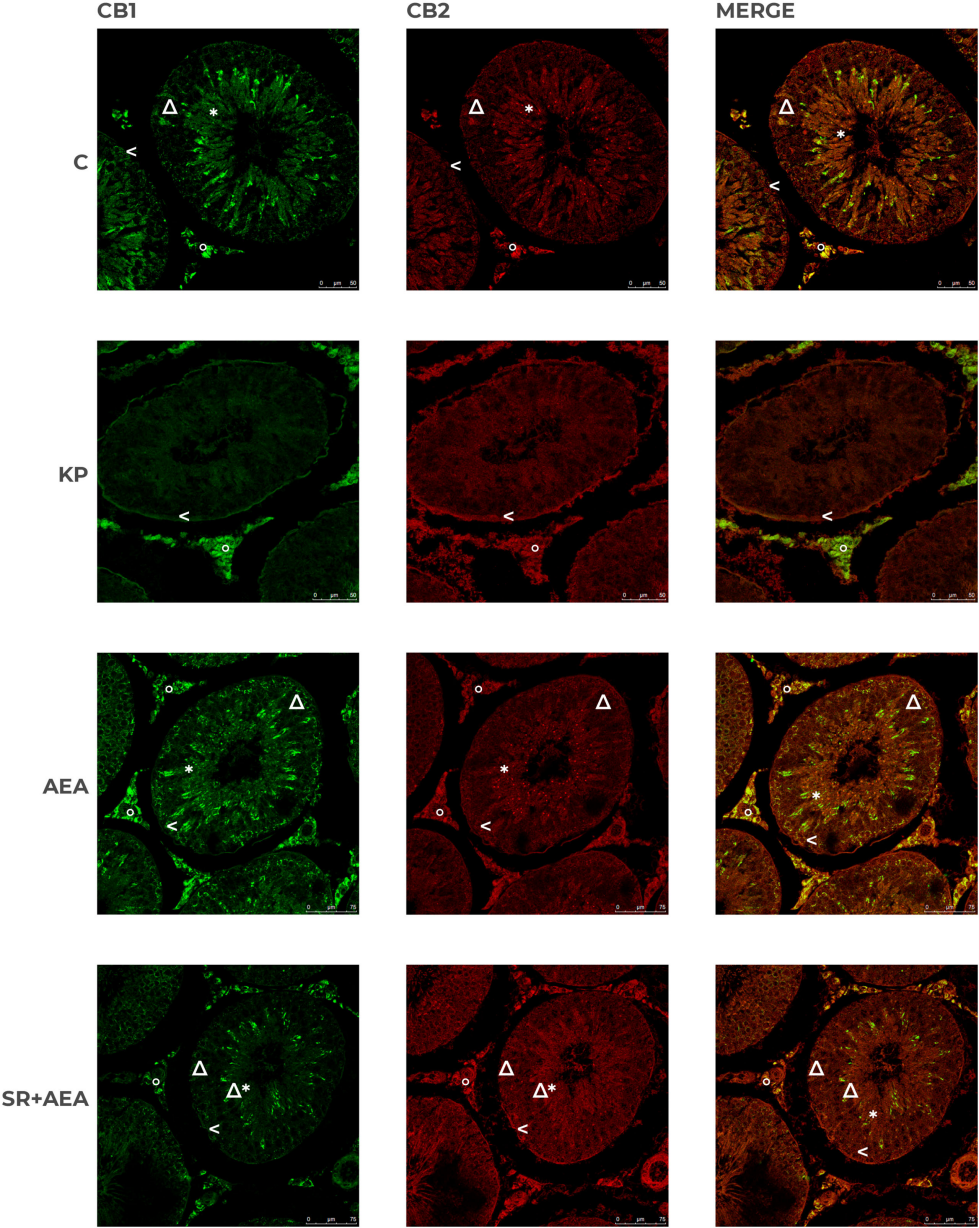
Distribution of CB1 and CB2 in rat testis treated with KP and AEA. Double immunofluorescence of CB1 (green) and CB2 (red) expression in control (C), kisspeptin (KP), anandamide (AEA), and SR141716A plus AEA (SR + AEA)-treated animals (*n* = 4 for each group). Magnification = C and KP ×63; AEA and SR + AEA, ×40. * Elongated spermatids, ↑ spermatocytes, ^ spermatogonia, Δ Sertoli cells, ° interstitium.

In control animals, CB1 was localized in both somatic (i.e., interstitial Leydig cells and Sertoli cells) and germ cells (i.e., spermatogonia in the basal compartment and round/elongating spermatids in the luminal compartment). CB2 had a more scattered and diffuse localization within the interstitium and the germinal epithelium, particularly in Sertoli cells and germ cells at mitotic and meiotic stages. CB1 and CB2 merge revealed a quite overlapping distribution (**Figure 9**). Following the KP treatment, CB1 was detectable in the interstitium and along the contour of the seminiferous tubule; a scattered and punctuate signal for CB2 was detectable in the germinal epithelium (**Figure 9**). In AEA-treated animals, CB1 and CB2 spotted signals were well evident in the interstitial and the germinal compartment, with overlapping signals (**Figure 9**). Pretreatment with rimonabant reduced CB1 but not CB2 staining in both interstitial and germinal compartments (**Figure 9**) corroborating the CB1-dependent action of AEA at the testicular level.

## Discussion

KS and ECS are two main signaling pathways in the central and peripheral control of reproduction (2–8), but their possible interrelation in the control of spermatogenesis and spermatogenesis progression has never been investigated in mammals. Currently, the only available data in the field are related to the control of spermatogenesis in the anuran amphibian *Pelophilax esculentus* (63), an experimental model used as an archetype for the study of master systems in the physiology of reproduction (64). Nevertheless, in this non-mammalian animal model, the reciprocal communication between KS and ECS occurs centrally to regulate the expression of *GnRH* and locally, via the estradiol synthesis and FAAH, to modulate the intratesticular tone of endocannabinoids, endocannabinoid signaling, and sex steroids responsible for spermatogenesis progression and maturation of post-meiotic stages (32, 33, 63). In mammals, few reports on the specific hypothalamic communication between KS and ECS are currently available. A gonadal cycle-dependent role of endocannabinoid signaling in the neuronal circuits involving Kiss1 neurons has been recently reported in female rats demonstrating that i) CB1 is expressed at the mRNA level in the GABAergic and glutamatergic subpopulations of Kiss1 neurons, ii) CB1 is localized in approximately 66% of the Kiss1 afferents to GnRH neurons, and iii) CB1 expression is modulated by estradiol (65). Despite data from females, studies on males are limited to the CB1-dependent decrease of *Kiss1* transcript in the medial preoptic area and ARC following acute stress, a condition that inhibits the HPG axis (66), and to the participation of hypothalamic CB1 in the disruption of reproductive axis by the inhibition of *Kiss1* mRNA and the increase of *gonadotrophin-inhibitory hormone* expression in a rat model of systemic infections (67).

Puberty is the endpoint of a maturational continuum that leads to reproductive competence and finds the main gatekeeper in the KS. In the present study, we demonstrated that in the hypothalamus of adolescent rats, Kp10 increased the expression of *GnRH* at the mRNA level as previously reported in vertebrates and cell lines (5, 6, 32, 63). Conversely, AEA, via CB1, modulated the KS, increasing Kiss1R and decreasing the corresponding ligand at the protein level, thus demonstrating the involvement of the ECS in the regulation of KS signaling. In mice, endocannabinoids tonically inhibit GABA(A)-R drive onto GnRH neurons (15, 68), and *CB1^−/−^* mice express lower *GnRH* mRNA level than wild type (17). Furthermore, the administration of AEA—*in vivo* by intracerebral injection or *ex vivo* in mediobasal hypothalamus explants—had negative effects on GnRH release following inflammation or alcohol (69, 70). These effects were reversed by sex steroids (13). In the present study, despite an increasing trend, the *in-vivo* AEA treatment was not significant for *GnRH* mRNA, contrary to previous data in non-mammalian species (14, 63). However, pretreatment with rimonabant, a drug acting as a CB1 antagonist, did not simply revert the AEA effect to the control level; instead, it seemed to exert an effect on itself by significantly reducing Kiss1R and CyP19 protein expression levels and inducing *GnRH* and Kiss1 in the ARC. This suggests that SR might also behave as a weak inverse agonist by binding receptors other than the CB1. Accordingly, it has been shown that in CB1-/- mice and human post-mortem brains, rimonabant could produce inverse agonist effects by binding further G-coupled receptors (mainly Gi3, Go, and Gz subtypes) (71). In addition, SR has been shown to function as a low-potency mixed agonist/antagonist of the CB1 and the transient receptor potential vanilloid receptor 1 (TRPV1) (4, 72, 73). This finding suggests the involvement of receptors other than CB1 in the complex neuronal network directly and indirectly controlling the activity of the HPG axis. In fact, the blockade of vanilloid receptors TRPV1 under basal or acute inflammatory conditions decreases *GnRH*, *Kiss1*, and *Rfrp3* (GNIH) at the mRNA level in male rats (74).

In the hypothalamus, Kiss1 is notably produced in a sex-specific manner by neuronal populations mainly located within the hypothalamic ARC (both females and males) and the anteroventral-peri-ventricular nucleus (AVPV) (females only) to modulate GnRH pulse and mediate sex steroid feedbacks (5, 6). The localization of Kiss1 and Kiss1R carried out in the present study confirmed that the ARC is the main brain region responsible for kisspeptin signaling in males, and the specific increase in the number of Kiss1+/Kiss1R+ neurons following Kp10 administration is consistent with the previously reported self-modulatory loop of the KS (31, 40, 75, 76). However, Western blot analysis of the basal hypothalamus did not show the same increase of Kiss1R found in the IHC of ARC. In addition, according to Western blot data, AEA significantly increased the number of Kiss1R+ cells in ARC, but the expression of Kiss1 protein decreased while remaining quite constant in the Western analysis. The apparent discrepancies in these results could be ascribable to the different tests used as Western blot was performed on total hypothalamic homogenates including all other nuclei, whereas immunostaining referred specifically to ARC. The fact remains that the ECS and KP systems strictly communicate with each other at the hypothalamic level, and the effects of AEA treatment are mediated by the CB1 receptor since its pharmacological inhibition by rimonabant attenuated the effects. Our results also showed that the co-treatment with SR141716 reduced Kiss1R (see Western blot data) and increased Kiss1 expression (IF analysis of ARC) to a higher extent than would be expected (i.e., results are significant compared with the control group). These findings suggest that the observed effects may be partially CB1 independent and could be mediated by other receptors such as TRPV1 (74, 77).

Notably, the present study revealed the Kiss1 localization along the ependyma of the lateral ventricle, and KP treatment exacerbated this condition. To the best of our knowledge, this is the first report showing the presence of Kiss1 in this brain region even though we were unable to discriminate if this accumulation represented the preferential route by which the administered Kp10 reached the brain during the treatment or was the result of an increase in endogenous Kiss1 as a result of positive feedback. This finding suggests additional roles for the peptide, including the homeostasis of the cerebrospinal fluid. Accordingly, a significant expansion of the ventricle area was observed in the brain of KP-treated animals in parallel to the increased expression of Cyp19, the enzyme that irreversibly converts testosterone into estradiol. Because estrogens are involved in post-natal brain development (4, 50), the enlargement of cerebral ventricles can be a sign of estrogenization in male rats (54); thus, the higher protein levels of Cyp19 might indicate an increase of estrogen- and estrogen-related signaling in the brain of KP-treated adolescent rats.

The exogenous modulation of the KS usually finds an endpoint in the altered levels of pituitary gonadotropins and sex steroids in plasma. Usually, kisspeptin (i.e., Kp10 or Kp54) administration increases gonadotropins and sex steroid production in vertebrates, including male rhesus monkeys, and in men and women as a consequence of HPG axis stimulation (5, 8, 45, 78–80). However, long-term KP treatment causes a desensitization of the HPG axis and reduces or has no effect on the production of sex steroids, and in some cases, it causes spermatogenesis damage impairing the physiology of Leydig cells, i.e., the intratesticular sources of testosterone [for a review, see (8, 45)]. In particular, Aytürk et al. reported opposite effects on testosterone biosynthesis following chronic or acute intraperitoneal administration of Kp10; the chronic administration was not effective, while the acute administration increased testosterone biosynthesis (59). Conversely, low circulating levels of both testosterone and 17β-estradiol characterize CB1-/- mice (16, 17). In the present study, Kp10 and AEA were administered chronically twice a week for 3 weeks, and both treatments did not have any significant effect in terms of the circulating levels of sex steroid and the intratesticular levels of 3βHsd and aromatase Cyp19. Conversely, KP treatment reduced *Cyp19* and *GnRH* mRNA, suggesting the existence of different modulatory mechanisms in the control of transcription and translation. Nevertheless, in both treatments, the circulating levels of Kiss1 did not differ from the control group, probably due to its short half-life, thus excluding the possible desensitization of the HPG axis in Kp10-treated animals. In addition, the testicular morphology did not reveal any anomaly following the KP treatment. Taken together, the measured hormonal values and the normal testis morphology exclude the previously reported acceleration of spermatogenesis due to the KP-dependent stimulation of the HPG axis [see (8) for a review]. Nevertheless, molecular markers of testis physiology were affected by KP administration. In fact, the testis expression of PCNA, a well-known marker of DNA replication occurring during the S phase of the cell cycle (60), was unchanged in KP-treated animals, whereas KP produced an increase in protein levels for both Lin28A and Sirt1. In this respect, Lin28A notably marks the spermatogonial progenitor population and regulates the cyclic expansion of the spermatogonial progenitor population as demonstrated by the reduced proliferation of spermatogonial stem cells in Lin28A knockout mice (81). Instead, Sirt1 is highly expressed from late spermatogonia onward with a role in acrosome biogenesis (82), and its expression level drops in response to environmental factors causing the impairment of the HPG axis and testis damage (62). Hence, these findings confirm the hypothesis that intratesticular KP signaling may represent a molecular switch for spermatogenesis progression in mammals, independently from testosterone biosynthesis, as demonstrated in co-cultures of spermatogonial cells with somatic cells (83), and differently from what happens in non-mammalian vertebrates (32, 33).

In this study, we also characterized the intratesticular KS in rats, currently a knowledge gap in the field, confirming previous reports in mammals that addressed Leydig cells, the main intratesticular source of Kiss1 and the main target for KS signaling [see (8, 45) for a review]. The present data revealed that the localization of Kiss1/Kiss1R in the germinal epithelium is quite scanty in the control testis and limited to a few elements in the basal and luminal compartments.

In contrast to the hypothalamus, in the testis, KP and AEA treatments got opposite effects on Kiss1R only, without any effect on Kiss1. In fact, Western blot revealed that Kp10 increased Kiss1R protein, whereas AEA, via CB1, decreased it. However, in the testis of Kp10-treated animals, both Kiss1 and Kiss1R immunoreactivity rates were significantly increased, not only in Leydig cells but also in pre-/early-meiotic stages. Conversely, the AEA treatment confined the localization of Kiss1 and Kiss1R just in pre-meiotic/meiotic spermatocytes and drastically reduced Kiss1/Kiss1R immunoreactivity in post-meiotic stages.

Furthermore, KP increased the intratesticular protein levels of CB1 and FAAH, the main AEA hydrolyzing enzyme; no effect was observed for CB2 or NAPE-PLD, the main enzyme in AEA biosynthesis. Hence, an intragonadic reciprocal crosstalk between the KS and ECS signaling occurs, thus suggesting that KP and AEA get direct effects in the gonad independently from the endocrine route responsible for the maintenance of spermatogenesis. The intratesticular localization of Kiss1, Kiss1R, CB1, and CB2 in the control and treated animals was confirmed by Western blot data, revealing that the KS and ECS systems are deeply involved in the intratesticular paracrine and autocrine communications between somatic cells and germ cells. Furthermore, KS had the ability to switch on a self-modulatory loop in both interstitial and germinal compartments to drive the progression of spermatogenesis and, in parallel, to direct AEA responsivity mainly to the interstitial compartment; conversely AEA switched off KS in post-meiotic stages and interstitium, directing Kiss1R signaling toward pre-/early-meiotic stages.

Lastly, in this study, we included the expression analysis of miRNAs related to the post-natal maturation of the brain and testis (i.e., *miR132*, *miR145*, *let-7a*, and *let7-b)* and the epigenetic modulation of pubertal transition (56, 61). In physiological conditions, *miR132*, *miR145*, and *let7-b* age-dependently increased from late infantile to puberty and reached the highest expression levels in the hypothalamus of adult rats (56). In contrast, in the testis, the expression levels of *let7-a*, *let7-b*, *miR132*, and *miR145* decreased from the neonatal-infantile stages to the transition from puberty to adulthood (61). Interestingly, KP and AEA via CB1 differently regulated puberty-related miRNAs in the brain and testis. In fact, this study revealed that the hypothalamic expression of *miR145* only resulted in a significant increase following AEA treatment; conversely, in the testis, all the analyzed miRNAs with the exception of *let7b* were significantly higher than controls in KP-treated animals and were significantly lower in the AEA-treated group. This point gets particular interest for further studies in the field. In fact, *miR-132* modulates ovarian and adrenal steroidogenesis (84) and promotes estradiol synthesis in granulosa cells (85), and its expression decreases in Sertoli cells following heat stress, a condition that heavily damages spermatogenesis (86). Furthermore, the increased expression of *miR145*, notably related to the progression of meiosis (61), parallels the increased levels of Lin28A protein, a marker of spermatogonial proliferation (81), in the testis of KP-treated animals. Consistently, no effect was observed on *let7b*, a testicular marker of meiotic and early post-meiotic stages (61), in all experimental conditions and in both the brain and testis.

**Figure 10:**
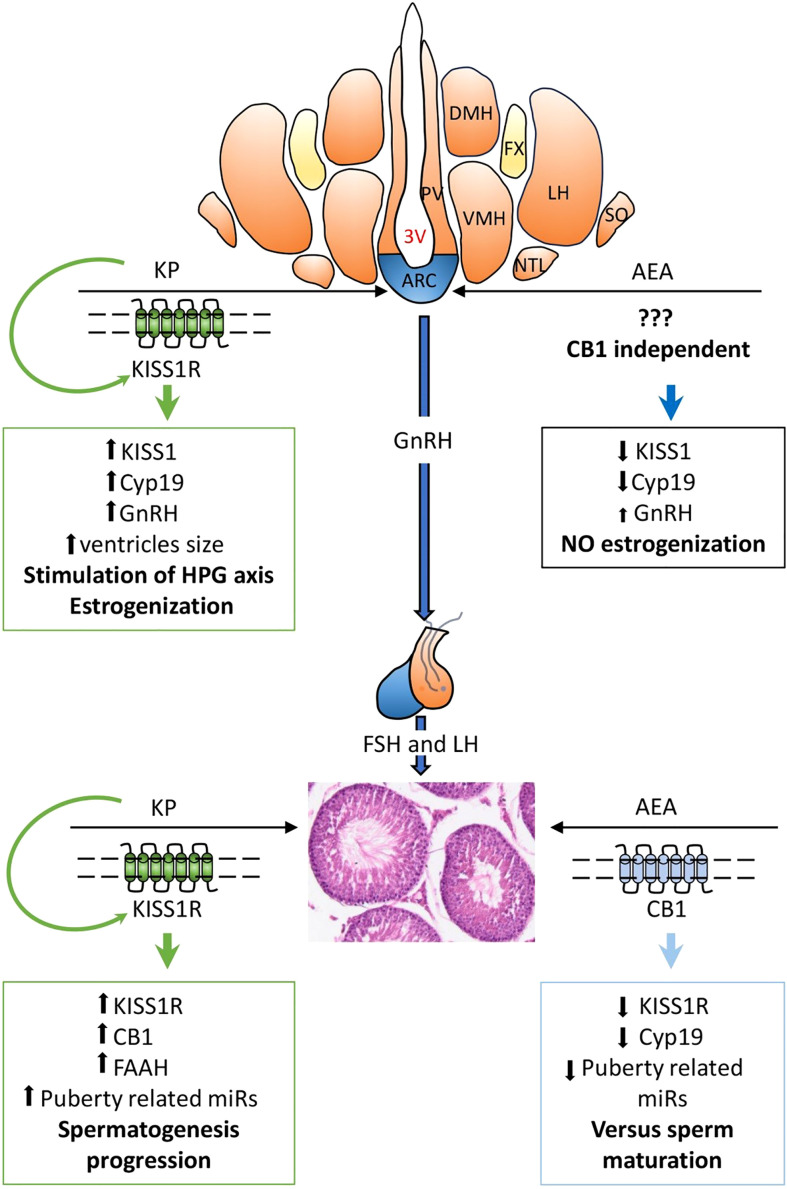
Main outcomes and functional hypotheses (bold font) of KPS and ECS stimulations and their interplay in regulating the hypothalamic–pituitary–gonadal (HPG) axis. AEA, anandamide; KP, kisspeptin; 3V, third ventricle; ARC, arcuate nucleus; DMH, dorsomedial hypothalamus; LH, lateral hypothalamus; NTL, hypothalamic lateral tuberal nucleus; PV, paraventricular nucleus; SO, supraoptic nucleus; VMH, ventromedial nucleus. GnRH, gonadotropin-releasing hormone; FSH, follicle-stimulating hormone; LH, luteinizing hormone. **↑**, increase; **↓**, decrease.

In conclusion, in the present study, we demonstrated that in both the hypothalamus and testis of adolescent rats, AEA modulates KS at the protein level, and that in the testis, KP affects CB1 and FAAH, the main regulator of AEA tone. A KP involvement in the progression of spermatogenesis is also suggested. A schematic representation of the main outcomes and functional interplay of KS and ECS stimulations along the HPG axis is reported in **Figure 10**.

Further investigations are certainly needed in the near future to clarify some points. The first limitation of this study concerns the real outcomes on gamete quality following the *in-vivo* modulation of KS/ECS. The additional limitations are to clearly identify which effects are hypothalamus-mediated and which are direct intragonadic effects of the tested drugs, together with the definition of the signaling pathway in KS/ECS crosstalk. In this respect, *ex*-*vivo* treatments of mediobasal hypothalamus and testis with specific agonists and the corresponding antagonists or the treatments with kisspeptin agonist and CB1 antagonists and vice versa have to be taken into consideration in the next studies. Nevertheless, the main strength of this work is the determination of a crosstalk between the two systems under examination, not only at the central level, but also for the first time in mammals, at the gonadal level. Taking into consideration the possible clinical relevance of KS and ECS in the treatment of infertility and *in-vitro* fertilization, the present data provide new molecular mechanisms and targets in the intricate communication network controlling male reproduction at the central and peripheral levels.

## Data availability statement

The original contributions presented in the study are included in the article/supplementary material. Further inquiries can be directed to the corresponding author.

## Ethics statement

The animal study was approved by the Ethical Committee of the University of Salerno and the Italian Ministry of University and Research (authorization no. 66/2020-PR dated 29/01/2020). The study was conducted in accordance with the local legislation and institutional requirements.

## Author contributions

MM: Investigation, Writing – original draft. RD’A: Formal Analysis, Investigation, Writing – original draft. EM: Investigation, Writing – original draft. GP: Investigation, Writing – review & editing. PD: Formal Analysis, Investigation, Writing – review & editing. SD’A: Investigation, Writing – original draft. NR: Investigation, Visualization, Writing – review & editing. FO: Writing – review & editing. CV: Writing – review & editing. SF: Formal Analysis, Writing – review & editing. RP: Formal Analysis, Writing – review & editing. AV: Conceptualization, Formal Analysis, Funding acquisition, Writing – original draft. RM: Conceptualization, Formal Analysis, Funding acquisition, Supervision, Writing – original draft. AS: Conceptualization, Formal Analysis, Writing – original draft, Supervision.
